# Admission systemic immune-inflammatory index predicts long-term mortality in patients with acute ischemic stroke: a retrospective analysis of the MIMIC-III database

**DOI:** 10.1186/s40635-026-00878-5

**Published:** 2026-03-09

**Authors:** Xiao Su, Xiuqing Tong, Ning Li, Boran Wang, Wei Gao, Yanmo Wang

**Affiliations:** 1 Department of Neurology, The Affiliated Hospital of Inner Mongolia Medical University, Hohhot, Inner Mongolia China; 2 Department of International Medicine, Inner Mongolia Autonomous Region People’s Hospital, Hohhot, Inner Mongolia China; 3 Department of Ultrasound Medicine, Inner Mongolia Medical College Affiliated Hospital, Hohhot, Inner Mongolia China; 4 Department of Medical Imaging, Inner Mongolia Medical College Affiliated Hospital, Hohhot, Inner Mongolia China; 5 Department of nuclear medicine, Inner Mongolia Medical University Cancer Hospital, Hohhot, Inner Mongolia China

**Keywords:** Biomarker, AISI, Acute ischemic stroke, Mortality, Intensive care units, MIMIC-III

## Abstract

**Background:**

The Aggregate Index of Systemic Inflammation (AISI) is a novel index based on platelets, neutrophils, and lymphocytes associated with the prognosis of patients with cancer and infectious diseases. However, its application in acute ischemic stroke (AIS) has rarely been reported. This study evaluated stroke prognosis using AISI by examining the relationship between levels of systemic immunoinflammatory indices at admission and patient outcomes at different times after onset.

**Methods:**

This was a retrospective cohort study. The data from 1222 stroke patients were obtained from multiparameter intelligent monitoring in the Intensive Care III database (MIMIC-III). Cox proportional risk model was conducted to estimate the relation between AISI, all-cause mortality, and ischemic. The findings were further validated with restricted cubic spline (RCS) and subgroup analyses.

**Results:**

A total of 1222 patients with AIS were classified into tertiles based on AISI levels, tertile 1: low AISI, AISI levels less than 3881 (*n* = 408), tertile 2: medium AISI, AISI levels 3881 to 8354 (*n* = 408), and tertile 3: high AISI, AISI levels greater than 8354 (*n* = 407). After adjusting for multiple covariates, it was found that AISI was related to all-cause mortality in stroke patients. Patients with high AISI had a 31% increased risk of death after 90 days (HR = 1.31, 95% CI 1.03–1.67, *P* = 0.03) compared to patients with low AISI. Patients with high AISI had a 27% increased risk of death after 365 days (HR = 1.27, 95% CI 1.03–1.58, *P* = 0.029) than low AISI patients. Furthermore, compared with patients with low AISI, patients with high AISI had a 30% increased risk of death after two years (HR = 1.30, 95% CI 1.05–1.60, *P* = 0.014). During the 2-year follow-up period, the use of RCS showed that the mortality rate gradually increased with the increase of AISI value after 5841.5.

**Conclusion:**

Systemic immunoinflammatory indices are related to long-term adverse outcomes in patients with AIS. Therefore, AISI is a promising inflammatory index for predicting the long-term prognosis of stroke.

## Introduction

Stroke remains a major global health burden and is the second leading cause of death and disability worldwide [1, 2]. Acute ischemic stroke (AIS), which accounts for approximately 87% of all stroke cases, results in substantial mortality and long-term neurological impairment when timely treatment is delayed [3–5]. Beyond the primary ischemic injury, AIS elicits a pronounced systemic inflammatory response that contributes to secondary brain damage and influences clinical outcomes [6–8]. Consequently, identifying reliable diagnostic and prognostic biomarkers that reflect both the direct injury and the associated inflammatory response in AIS is essential for improving patient outcomes and guiding treatment strategies.

Peripheral blood cell–based inflammatory indices, such as the neutrophil-to-lymphocyte ratio (NLR), platelet-to-lymphocyte ratio (PLR), and systemic inflammatory response index (SIRI), have been widely investigated in various cardiovascular and inflammatory diseases [9–14]. Although these markers are readily available and clinically practical, their prognostic performance is limited by suboptimal sensitivity and specificity, partly because each captures only a narrow aspect of the complex immune response. For instance, NLR and PLR focus on single proportional relationships, while SIRI incorporates neutrophils and monocytes but omits platelets, an essential contributor to thrombosis and post-stroke inflammation [15, 16]. As a result, these indices may fail to fully represent the interplay between acute inflammation, chronic immune activation, thrombogenesis, and post-stroke immunosuppression, all of which influence long-term outcomes [17, 18].

The Aggregate Index of Systemic Inflammation (AISI), calculated using neutrophil, monocyte, platelet, and lymphocyte counts, has recently been proposed as a more comprehensive measure of systemic inflammatory status. Although AISI has shown prognostic value in several nonstroke populations, evidence regarding its clinical utility in AIS remains scarce. In particular, whether AISI can serve as a reliable marker for long-term mortality after ischemic stroke has not been systematically evaluated.

Therefore, this study aimed to investigate the association between AISI and long-term mortality in patients with AIS using the MIMIC-III database, which provides high-quality longitudinal clinical data suitable for prognostic research.

## Methods

### Data source

The data were obtained from the Intensive Care III database version 1.4 (MIMIC-IIIv1.4), a publicly available single-center critical care database. The Institutional Review Boards of Beth Israel Deaconess Medical Center and the Massachusetts Institute of Technology (Cambridge University) approved this project. Patient information in this database was anonymized to protect the privacy of the patients. All patients signed informed consent. MIMIC-III contains information on 46,520 patients admitted to the Beth Israel Deaconess Medical Center in Boston, Massachusetts, from 2001 to 2012 [19]. Information files comprised population chart events such as demographics, vital signs, lab tests, fluid balance, and vital status. Hospital staff recorded hourly physiological data from the International Classification of Diseases, Ninth Revision (ICD-9) code records verified by nurses on bedside monitors at patient discharge. The database contained a written assessment of the corresponding period of radiology films by specialists. The documentation in the database was provided by clinicians, data scientists, information personnel, and users. An online training course was required prior to accessing the database. Su Xiao passed the exam to protect human research participants and was given access to the database (certification number: 45610476) for data extraction.

### Patient selection

Patients over 18 with AIS were chosen using the International Classification of Diseases (ICD)-9 code. The following exclusion criteria were applied to this study: (1) patients with a history of transient ischemic attack (TIA) that did not progress to AIS; (2) patients without leukocyte, neutrophil, lymphocyte, platelet, and monocyte data collected within the first 24 h of admission; and (3) patients with inflammatory diseases or active infections, such as rheumatoid arthritis, systemic lupus erythematosus, inflammatory bowel disease, sepsis, acute respiratory infections, exacerbation of chronic obstructive pulmonary disease, active viral infections, and chronic hepatitis. If patients were admitted multiple times, data were collected only for the first admission.

### Data extraction

The data were retrieved from the MIMIC-III database using Structured Query Language (SQL) and PostgreSQL software (version 9.6). The data encompassed demographic parameters, vital signs, laboratory parameters, comorbidity parameters, date of death, date of admission, and other variables. Comorbidities included hypertension, coronary atherosclerotic heart disease, dyslipidemia, and diabetes mellitus. Laboratory data, including activated partial thromboplastin time (APTT), prothrombin time (PT), creatinine, international normalized ratio (INR), platelets, lactate, red blood cell distribution width (RDW), white blood cells (WBC), lymphocytes, neutrophil, platelet, mononuclear cell, red blood cells (RBC), HDL, LDL, TC, TG, urea, the concentration of sodium, potassium, magnesium, chloride, and phosphate, were extracted from blood samples collected within the first 24 h following hospital admission. We also assessed the Glasgow Coma Scale (GCS), which reflects the degree of neurological deficit. We also collected data on whether patients were treated with thrombolysis. Vital signs such as diastolic blood pressure (DBP), systolic blood pressure (SBP), and heart rate were obtained. All variables, including demographic parameters, vital signs, laboratory measurements, comorbidity data, GCS, and other variables such as reperfusion therapy, were collected within 24 h of admission. To address missing data points, we utilized multiple imputation methodology, a sophisticated statistical approach designed to mitigate potential bias and preserve the statistical integrity of the dataset. The sole endpoint of this study was all-cause mortality at 30 days, 90 days, 365 days, and 2 years from the date of hospital admission.

### Assessment of AISI

The Aggregate Index of Systemic Inflammation (AISI) was calculated using the following formula:

$$\mathrm{AISI}=\frac{\mathrm{Neutrophils}\times \mathrm{platelets}\times \mathrm{monocytes}}{\mathrm{lymphocytes}}.$$

AISI integrates four key immune components—acute inflammation (neutrophils), chronic inflammation (monocytes), thrombosis (platelets), and post-stroke immunosuppression (lymphocytes)—and is considered a more comprehensive indicator of systemic inflammation [20].

Because no validated clinical reference range or biological cutoff for AISI has been established in acute ischemic stroke (AIS), we used a data-driven tertile classification. This method is widely applied for composite inflammatory indices when prior thresholds are unavailable, as it minimizes bias from arbitrary cutpoints and preserves statistical efficiency by ensuring balanced group sizes [21]. Tertile-based grouping has also been adopted in previous AISI and SIRI investigations to evaluate dose–response associations across populations with differing inflammatory backgrounds [22–26]. Accordingly, patients were categorized into three tertiles based on the distribution of baseline AISI values in our cohort, with the lowest tertile (Q1) serving as the reference group.

### Statistical analysis

The mean ± standard deviation was taken for continuous variables if the data were normally distributed (SD). The median (one, three, and four quartiles) was derived if the data were not normally distributed. Kolmogorov–Smirnov test estimated whether the continuous data were normally distributed. Categorical variables were expressed as numbers or percentages. The Chi-square or Fisher’s exact test, one-way ANOVA, and Kruskal–Wallis H test determined significant differences between groups.

Cox proportional risk regression models analyzed potential associations between AISI and all-cause mortality at 30 days, 90 days, 365 days, and 2 years expressed as risk ratios (HRs) with 95% confidence intervals (CIs). Variables such as demographic characteristics, comorbidities, and laboratory tests were included as confounders in multivariate Cox regression analysis. Three multivariate models were used for each endpoint based on the AISI group, identifying the first subgroup as the reference. In model I, the following covariates were adjusted: age, race, sex, hypertension, coronary atherosclerotic heart disease, dyslipidemia, and diabetes mellitus. In model II, the following were accustomed: thrombolytic agent, creatinine, lactate, urea, RDW, red blood cells, potassium, phosphate, chloride, magnesium, sodium, APTT, PT, INR, TC, TG, HDL, LDL, SBP, DBP, and heart rate. Model III was further adjusted for the Glasgow Coma Scale (GCS) score.

To explore the potential nonlinear association between AISI and mortality and to identify a biologically informative threshold, restricted cubic spline (RCS) analysis was performed based on the fully adjusted Cox proportional hazards model (Model III). The RCS model was fitted with 5 knots placed at the 5th, 35th, 50th, 65th, and 90th percentiles of the AISI distribution.

Subgroup analysis was performed using a Cox proportional risk regression model. *P* < 0.05 was defined as statistically significant in this analysis. All the analyses were conducted using Stata software (version 16.0, CRAN) and SPSS software (v22.0; IBM, Armonk, NY), and *P* < 0.05 was defined as statistically significant. OriginPro 2019b plotted the histogram of mortality, and R version 4.1.3 was used to plot the forest map.

## Results

### Subject characteristics

**Fig. 1:**
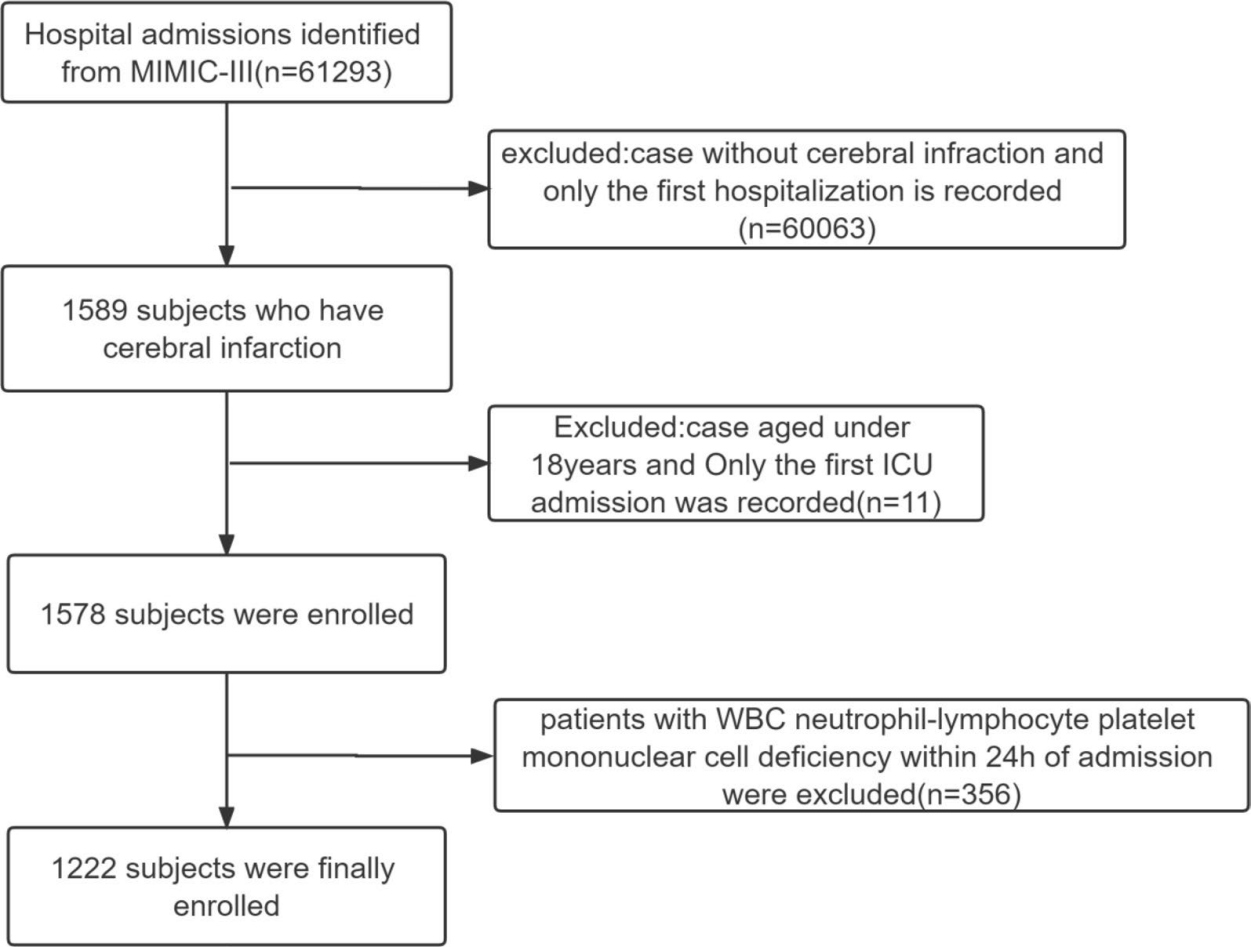
Flow diagram of selection for patients. This flowchart illustrates the selection of acute ischemic stroke (AIS) patients from the MIMIC-III database. Patients were initially screened based on an AIS diagnosis, and exclusions were applied for age < 18 years, missing baseline laboratory parameters required for AISI calculation (neutrophils, monocytes, lymphocytes, or platelets). The final cohort included patients who had complete baseline data within 24 h of admission and available follow-up outcomes for mortality analyses

As per the selection criteria mentioned above, 1222 patients with AIS were included in this study (Fig. 1). Patients were classified into tertiles based on AISI levels, tertile 1: low AISI, AISI levels less than 3881 (*n* = 408), tertile 2: medium AISI, AISI levels 3881 to 8354 (*n* = 408); and tertile 3: high AISI, AISI levels greater than 8354 (*n* = 407), and the basic information is listed in Table 1. Compared with the other two groups, patients with AISI > 8354 had a history of hyperlipidemia, higher levels of DBP, SBP, heart rate, APTT, creatinine, INR, PT, RBC, RDW, urea, and sodium (overall *P* < 0.05).

**Table 1:** Baseline characteristics of the study patients according to admission Aggregate Index of Systemic Inflammation (AISI) tertiles

Characteristics	AISI			*P*-value
	≤ 3881 (n = 408)	> 3881, ≤ 8354 (n = 407)	> 8354 (n = 407)	
Case, n (%)				
Age (years), mean (SD)	68.85 (16.55)	67.65 (16.36)	69.57 (15.50)	0.235
Gender, n(%)				
Female	227 (55.6%)	197 (48.4%)	208 (51.1%)	0.113
Male	181 (44.4%)	210 (51.6%)	199 (48.9%)	
Ethnicity, n(%)				0.007
1 = WHITE	283 (69.4%)	289 (71.0%)	308 (75.7%)	
2 = BLACK	55 (13.5%)	36 (8.8%)	25 (6.1%)	
3 = OTHER	70 (17.2%)	82 (20.1%)	74 (6.1%)	
Vital signs, mean (SD)				
Heart rate	81.09 (16.15)	81.97 (16.03)	85.71 (16.15)	< 0.001
Systolic BP(mmHg)	131.98 (19.96)	132.90 (19.54)	128.33 (20.50)	0.003
Diastolic BP(mmHg)	63.91 (11.46)	65.91 (12.32)	63.60 (11.61)	0.01
Risk factors, n (%):				
Hypertension	240 (58.8%)	246 (60.4%)	222 (54.5%)	0.212
Diabetes	139 (34.1%)	133 (32.7%)	125 (30.7%)	0.59
Hyperlipidemia	173 (42.4%)	163 (40.0%)	120 (29.5%)	< 0.001
Coronary atherosclerotic heart disease	98 (24.0%)	102 (25.1%)	94 (23.1%)	0.806
Thrombolytic agent	42 (10.3%)	37 (9.1%)	42 (10.3%)	0.799
GCS(SD)	12.75 (3.09)	12.68 (3.25)	12.52 (3.26)	0.579
Laboratory items, mean (SD)				
APTT	26.60 (24.000–30.90)	26.40 (23.80–30.30)	27.30 (23.90–32.60)	0.017
Creatinine	1.00 (0.80–1.40)	1.00 (0.80–1.30)	1.00 (0.80–1.50)	< 0.001
Glucose	139.45 (62.25)	152.86 (84.14)	156.19 (66.89)	0.026
TC, mmol/L	153.29 (183.47)	138.67 (158.63)	153.44 (198.96)	0.409
TG, mmol/L	164.23 (44.54)	165.92 (46.88)	159.41 (16.45)	0.111
INR	1.10 (1.00–1.30)	1.10 (1.00–1.30)	1.20 (1.10–1.40)	0.002
Lactate	2.12 (1.94)	2.22 (2.14)	2.29 (1.87)	0.444
PT	13.10 (12.40–14.50)	13.20 (12.40–14.40)	13.50 (12.70–15.20)	0.004
RBC	4.13 (0.80)	4.26 (0.65)	4.15 (0.81)	0.021
RDW	14.44 (1.78)	14.18 (1.63)	14.73 (1.86)	< 0.001
Urea	20.00 (15.00–29.00)	19.00 (14.00–27.00)	22.00 (15.00–34.00)	< 0.001
NA	139.46 (3.86)	139.35 (4.42)	138.71 (4.54)	0.054
K	4.21 (0.78)	4.23 (0.77)	4.31 (0.88)	0.162
Mg	1.99 (0.36)	1.94 (0.33)	1.96 (0.40)	0.179
HDL	44.06 (15.07)	43.98 (15.85)	43.81 (16.76)	0.975
LDL	94.00 (40.14)	90.48 (38.50)	89.35 (38.67)	0.208
30-day mortality,n(%)	107/408 (26.2%)	91/407 (22.4%)	133/407 (32.7%)	0.004
90-day mortality,n(%)	121/408 (29.7%)	120/407 (29.5%)	170/407 (41.8%)	< 0.001
365-day mortality,n(%)	159/408 (39.0%)	148/407 (36.4%)	209/407 (51.4%)	< 0.001
2-Year mortality,n(%)	174/408 (42.6%)	161/407 (39.6%)	227/407 (55.8%)	< 0.001

Data are presented as mean (standard deviation), median (interquartile range), or number (percentage). Normality of continuous variables was assessed using the Kolmogorov–Smirnov test. One-way analysis of variance (ANOVA) was used for normally distributed continuous variables across the three groups, and the Kruskal–Wallis H test was applied for non-normally distributed continuous variables. Categorical variables were compared using the Chi-square test or Fisher's exact test, as appropriate. The reported *P* values refer to the overall comparison among the three AISI groups. In the subsequent Cox proportional hazards regression analyses, the lowest AISI tertile (≤ 3881) was treated as the reference group

*AISI* Aggregate Index of Systemic Inflammation, *SD* standard deviation, *BP* blood pressure, *GCS* Glasgow Coma Scale, *APTT* activated partial thromboplastin time, *INR* international normalized ratio, *PT* prothrombin time, *RBC* red blood cell, RDW red cell distribution width, *TC* total cholesterol, *TG* triglyceride, *HDL* high-density lipoprotein cholesterol, *LDL* low-density lipoprotein cholesterol

### AISI levels and all-cause mortality

**Fig. 2:**
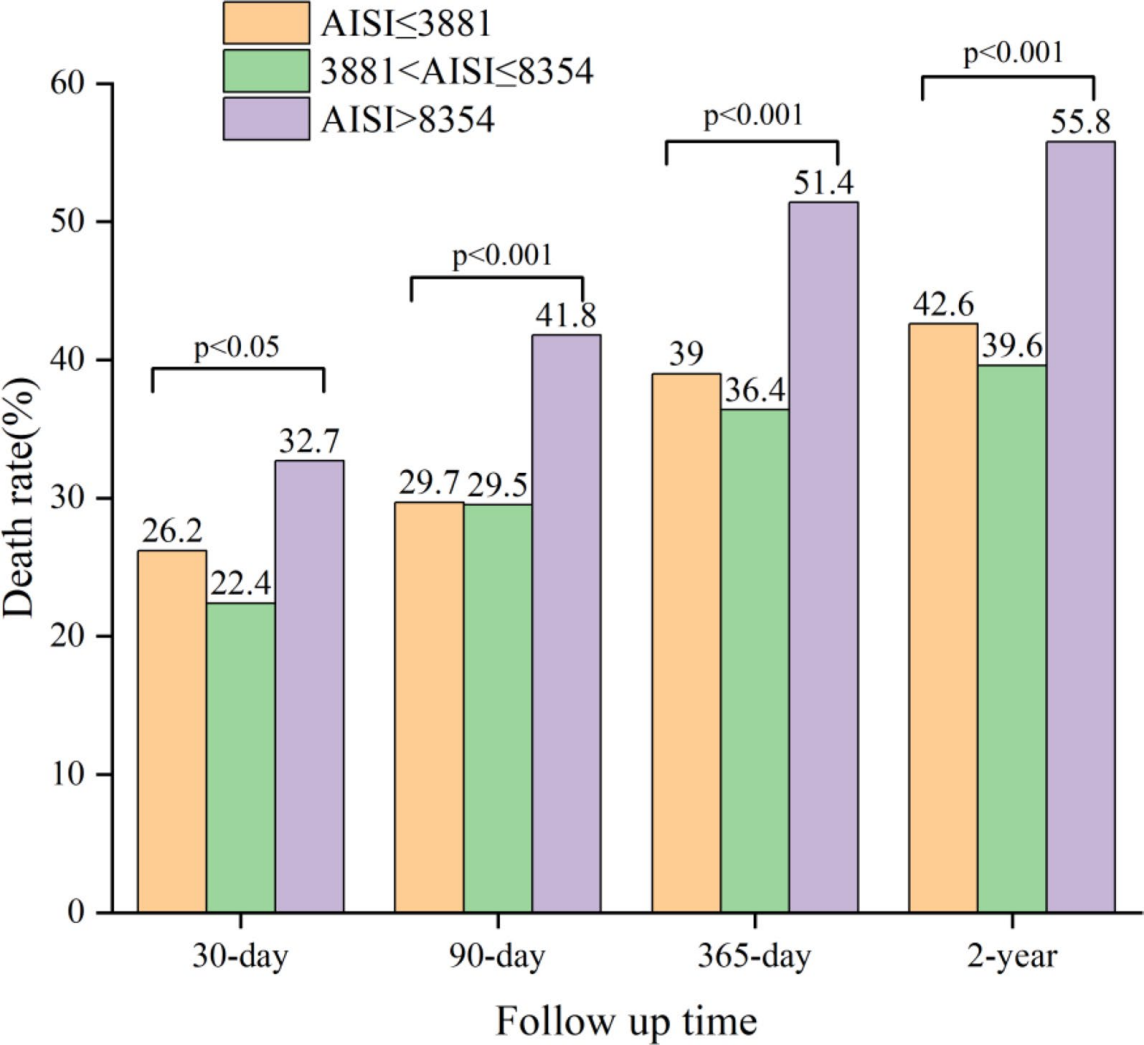
Mortality rates at different follow-up time points by admission AISI tertiles. The graph compares all-cause mortality among acute ischemic stroke patients stratified into low (AISI ≤ 3,881), intermediate (3,881 < AISI ≤ 8,354), and high (AISI > 8,354) AISI groups at 30 days, 90 days, 365 days, and 2 years. Group-specific mortality rates are annotated. *P* values indicate significant differences across AISI groups at each time point (overall *P* < 0.05)

**Fig. 3:**
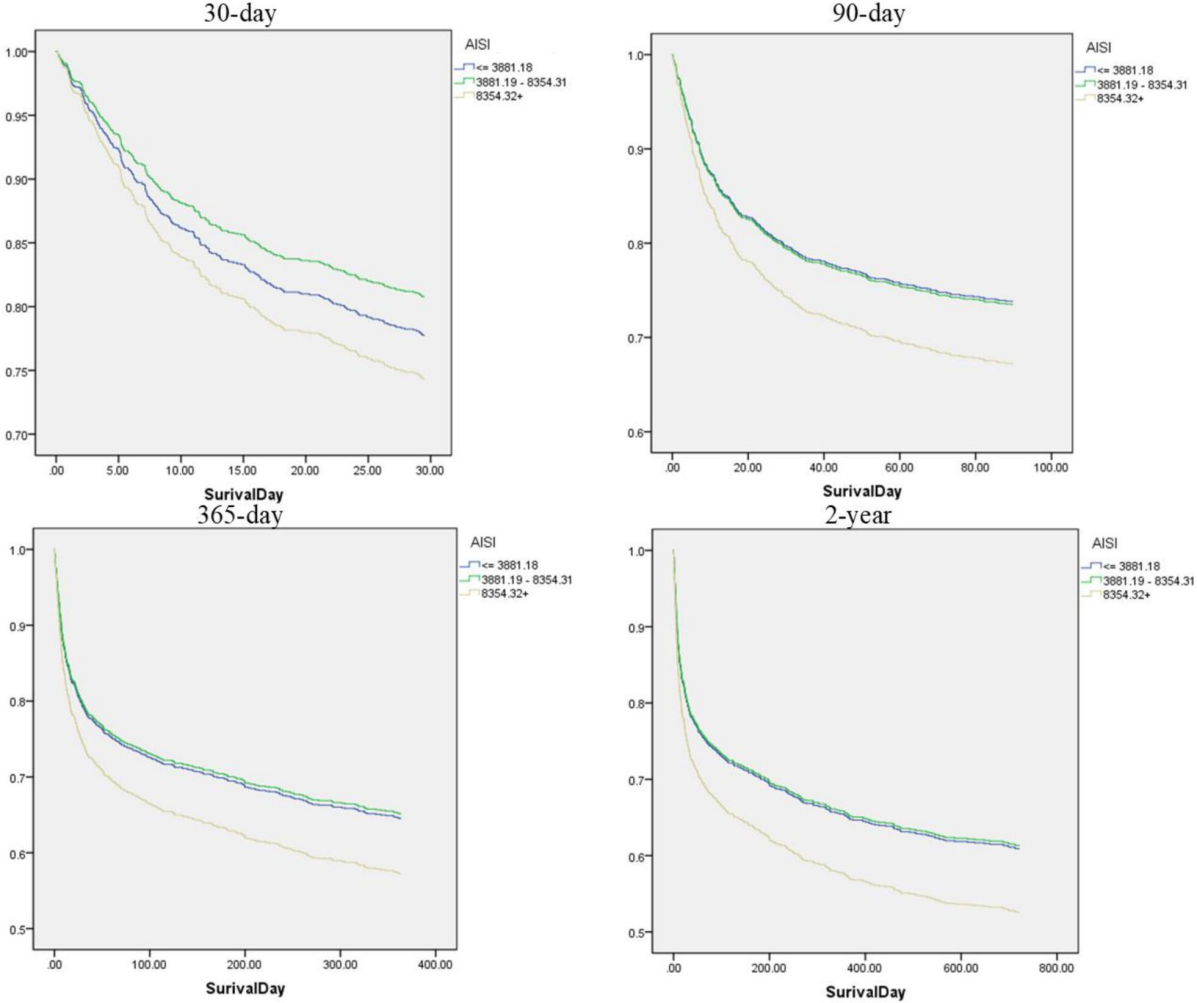
Survival probability curves by admission AISI levels across different follow-up periods. (**a**) 30-day, (**b**) 90-day, (**c**) 1-year, and (**d**) 2-year survival curves are shown for patients with acute ischemic stroke stratified into low (AISI ≤ 3881), intermediate (3881–8354), and high (AISI ≥ 8354) tertiles of the Aggregate Index of Systemic Inflammation (AISI). The curves represent adjusted survival probabilities derived from the fully adjusted Cox proportional hazards model (Model III), with time (days) on the x-axis and survival probability on the y-axis

The outcome of interest in this study was all-cause mortality, assessed at 30 days, 90 days, 365 days, and 2 years. As shown in Fig. 2, mortality differed significantly among the low, medium, and high AISI groups at each time point, indicating that patients with different AISI levels had statistically distinct risks of death during follow-up. Therefore, AISI may serve as a potential biomarker for prognosis in patients with acute ischemic stroke. The Cox proportional hazards regression models were used to determine different AISI levels and all-cause mortality at 30 days, 90 days, one year, and two years in patients with acute ischemic stroke (Table 2, Fig. 3). The lower AISI group (AISI ≤ 3881.18) was used as a reference; high AISI was related to an increased risk of all-cause mortality at 30 days, 90 days, 365 days, and two years in the non-adjusted model (*P* for trend < 0.05). In model I, there was no link between AISI and 30-day all-cause mortality after adjusting for age, race, sex, hypertension, CVD, dyslipidemia, diabetes, and high AISI was associated with 90-day, 365-day, and 2-year all-cause mortality was related with an increased risk of mortality (*P* for trend < 0.05). After further adjustment for thrombolytic agent, creatinine, lactate, urea, RDW, red blood cells, potassium, phosphate, chloride, magnesium, sodium, APTT, INR, TC, TG, HDL, LDL, SBP, DBP, and heart rate in model II following observations were made. There was no relationship between AISI and 30-day all-cause mortality, and high AISI was significantly associated with mortality and longer follow-up (*P* for trend < 0.05). Subsequently, with further adjustment for GCS in model III, no relationship was found between AISI and 30-day all-cause mortality, and high AISI was significantly associated with mortality with longer follow-up (90 days: HR = 1.31, 95% CI 1.03–1.67, 365 days: HR = 1.27, 95% CI 1.03–1.58 and two years: HR = 1.30, 95% CI 1.05–1.60).

**Table 2:** HRs (95% CIs) for all-cause mortality across groups of AISI

	Non-adjusted		Model I		Model II		Model III	
Variable	HR (95% CIs)	*P * value	HR (95% CIs)	*P * value	HR (95% CIs)	*P * value	HR (95% CIs)	*P * value
30-day all-cause mortality								
AISI								
< = 3881	1.0 (ref)		1.0 (ref)		1.0 (ref)		1.0 (ref)	
3381–8354	0.83 (0.63,1.09)	0.182	0.85 (0.64,1.12)	0.252	0.87 (0.66,1.16)	0.351	0.85 (0.64,1.13)	0.263
> 8354	1.31 (1.01,1.68)	0.040	1.22 (0.94,1.58)	0.134	1.16 (0.89,1.50)	0.285	1.18 (0.90,1.54)	0.227
*P* trend		0.040		0.134		0.285		0.227
90-day all-cause mortality								
AISI								
< = 3881	1.0 (ref)		1.0 (ref)		1.0 (ref)		1.0 (ref)	
3381–8354	0.96 (0.75,1.24)	0.775	0.99 (0.77,1.27)	0.27	1.04 (0.80,1.34)	0.787	1.01 (0.78,1.31)	0.916
> 8354	1.50 (1.19,1.90)	0.001	1.37 (1.08,1.73)	0.01	1.29 (1.01,1.29)	0.042	1.31 (1.03,1.67)	0.030
*P* trend		0.001		0.01		0.042		0.030
365-day all-cause mortality								
AISI								
< = 3881	1.0 (ref)		1.0 (ref)		1.0 (ref)		1.0 (ref)	
3381–8354	0.91 (0.723,1.14)	0.394	0.94 (0.75,1.17)	0.43	0.99 (0.78,1.24)	0.839	0.98 (0.78,1.23)	0.852
> 8354	1.45 (1.18,1.78)	0.001	1.32 (1.07,1.62)	0.01	1.24 (1.00,1.53)	0.050	1.27 (1.03,1.58)	0.029
*P* trend		0.001		0.01		0.050		0.029
2-year all-cause mortality								
AISI								
< = 3881	1.0 (ref)		1.0 (ref)		1.0 (ref)		1.0 (ref)	
3381–8354	0.90 (0.73,1.12)	0.337	0.94 (0.75,1.16)	0.541	0.99 (0.79,1.24)	0.981	0.97 (0.79,1.23)	0.901
> 8354	1.45 (1.19,1.77)	0.001	1.33 (1.09,1.63)	0.005	1.26 (1.02,1.54)	0.031	1.30 (1.05,1.60)	0.014
*P* trend		0.001		0.005		0.031		0.014

Models were derived from Cox proportional hazards regression models. Non-adjusted model, adjusted for none. Adjust I model, adjusted for age, race, sex, hypertension, cardiovascular disease, dyslipidemia, and diabetes mellitus. Adjust II model, adjusted for age, ethnicity, gender, hypertension, cardiovascular disease, dyslipidemia, diabetes, APTT, chloride, creatinine,INR,lactate,Mg,phosphate,K,PT,RBC,RDW,NA,urea,TC,TG.HDL.LDL,systolic blood pressure, diastolic blood pressure, heart rate and thrombolytic agent infusion therapy. Model III further adjusted for Glasgow Coma Score (GCS)

*HR* hazard ratio, *CI* confidence interval

### Restricted cubic spline analysis (RCS): exploring the biological threshold of AISI for mortality risk

**Fig. 4:**
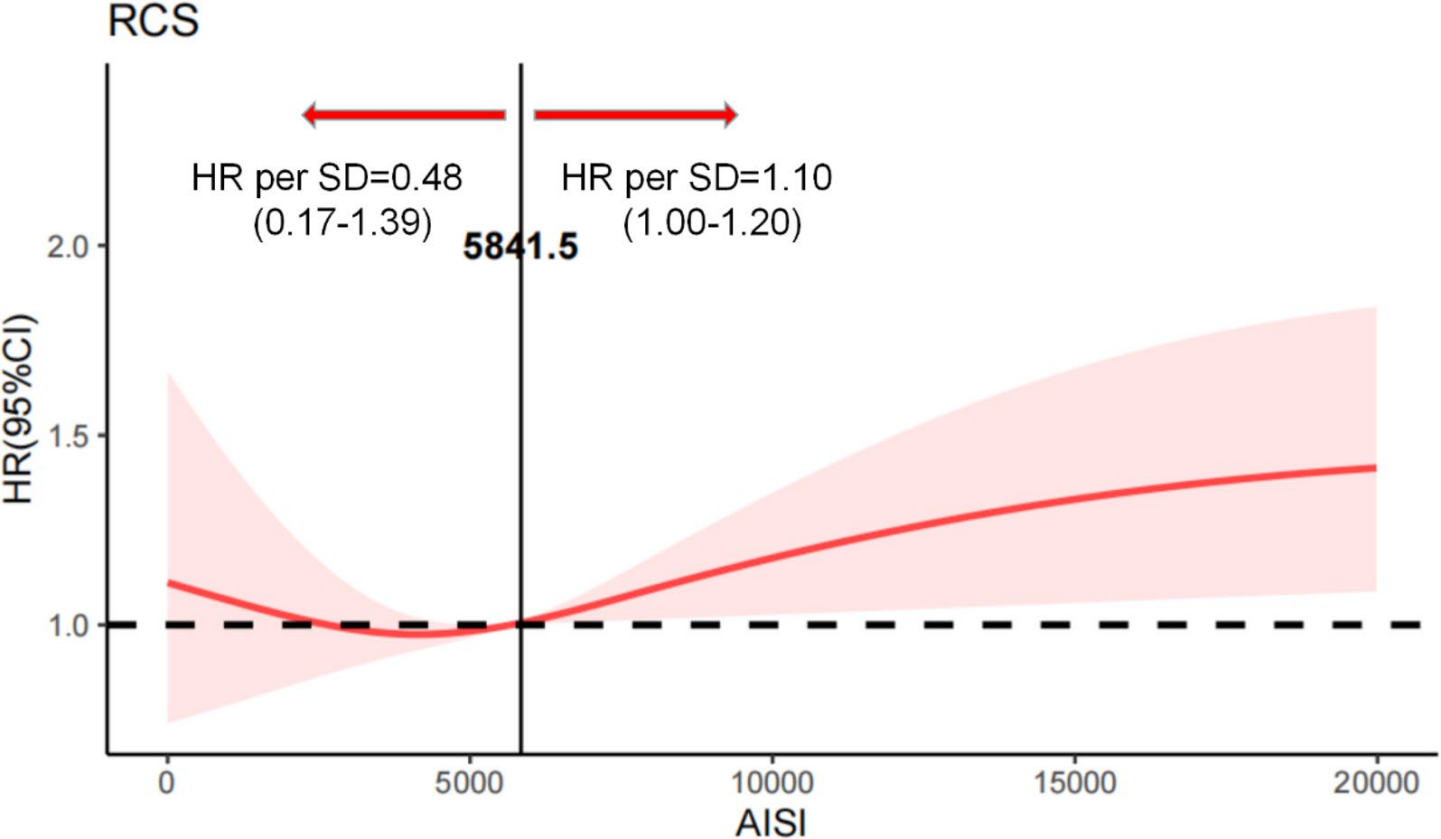
Association between AISI and all-cause mortality using a restricted cubic spline regression model. Results were adjusted for age, race, sex, hypertension, cardiovascular disease, dyslipidemia, diabetes mellitus, Thrombolytic agent, creatinine, lactate, urea, RDW, red blood cells, potassium, phosphate, chloride, magnesium, sodium, APTT, PT, INR, TC, TG, HDL, LDL, systolic blood pressure, diastolic blood pressure, and Glasgow coma score (i.e., adjusted according to Model III). Restricted cubic spline regression model was conducted with 5 knots at the 5th, 35th, 50th, 65th, and 90th percentiles of AISI. The dotted lines represent the 95% confidence intervals for the spline model (reference is 5841.5). HR indicates hazard ratio. Below the AISI value of 5841.5, the hazard ratio per standard deviation higher predicted all-cause mortality was 0.48 (0.17 to 1.39). Above the AISI value of 5841.5, the hazard ratio per standard deviation higher predicted all-cause mortality was 1.10 (1.00 to 1.20)

To explore the relationship between AISI and 2-year all-cause mortality, we conducted a restricted cubic spline (RCS) analysis based on the fully adjusted Cox proportional hazards model (Model III). As shown in Fig. 4, the spline curve suggested a U-shaped pattern between AISI and mortality risk after adjustment for all prespecified confounders. Specifically, the estimated hazard appeared to decrease at lower AISI levels and reached a nadir at an AISI value of approximately 5841.5, followed by a gradual increase in risk with higher AISI values. Below this point, each standard deviation increase in AISI was associated with a lower hazard of all-cause mortality (HR = 0.48, 95% CI 0.17–1.39), although this association did not reach statistical significance. In contrast, above an AISI value of 5841.5, higher AISI levels were associated with a trend toward increased mortality risk per standard deviation increase (HR = 1.10, 95% CI 1.00–1.20). Overall, these findings suggest a nonlinear, U-shaped association between AISI and long-term mortality risk. The estimated minimum AISI value of approximately 5841.5 may be considered an exploratory biological reference point for this nonlinear pattern, rather than a definitive clinical threshold.

### Subgroup analysis

**Fig. 5:**
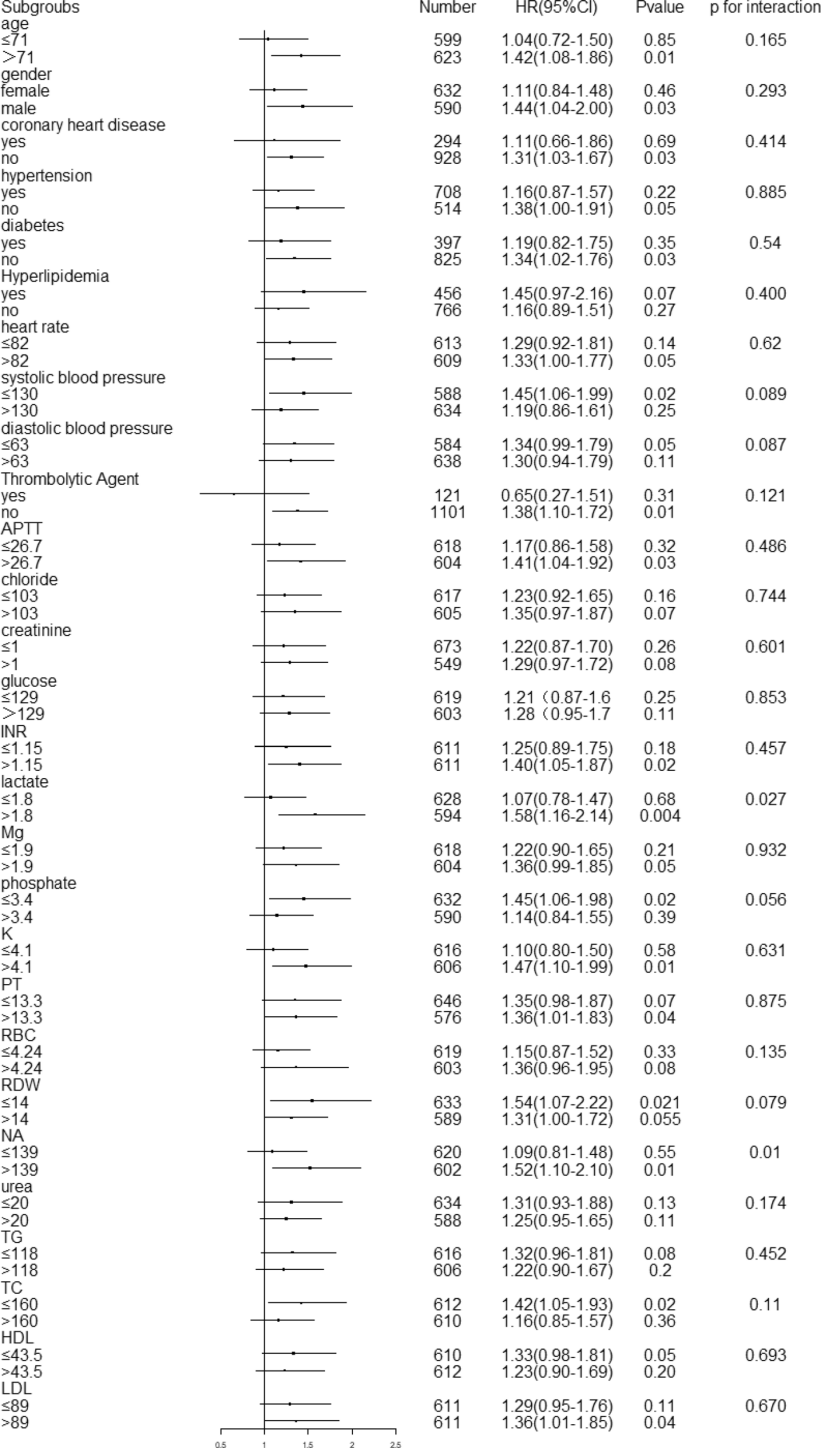
Subgroup analysis for the association between admission AISI and 2-year all-cause mortality. Forest plot illustrating hazard ratios (HRs) and 95% confidence intervals (CIs) across various subgroups, stratified by demographic characteristics, comorbidities, key laboratory parameters, and thrombolysis status. The reference group for AISI is the low tertile (AISI ≤ 3881). Subgroup-specific HRs (boxes) and 95% CIs (horizontal lines) are shown. P values for interaction (P-interaction) are provided for each subgroup. *AISI* Aggregate Index of Systemic Inflammation, *HR* hazard ratio, *CI* confidence interval

To elucidate potential heterogeneity, we performed comprehensive subgroup analyses stratified by demographic characteristics (age and sex), clinical comorbidities, baseline physiological parameters, therapeutic interventions (thrombolysis status), and key laboratory indicators. Subgroup analyses of laboratory parameters and comorbidities were performed by level, as shown in Fig. 5. AISI levels were similar to 2-year all-cause mortality in most strata, and there was no interaction (*P* > 0.05). A significant interaction of erythrocytes and phosphate was observed. Patients with hyperlipidemia (*P* = 0.03) and blood sodium (*P* = 0.027) levels and lower SBP (*P* = 0.037) levels demonstrated potentially higher mortality. Subgroup analyses of stroke classification were performed by level, as shown in Table 3. Subgroup analyses of stroke classification were performed by level, as shown in Table 3. Only in large-artery atherosclerosis were high AISI levels associated with higher mortality; (HR = 1.38, 95% CI 1.03–1.85, *P* = 0.028), *p*-interaction 0.05.

**Table 3:** Subgroup analysis of associations between AISI and 2-year all-cause mortality based on different types of acute ischemic stroke

Subgroubs	Number	HR (95%CI)	*p*-value	*p* for interaction
Large artery atherosclerosis	686	1.38 (1.03–1.85)	0.028	0.05
Small vessel occlusion	26	3.68(0.29–46.79)	0.314	
Cardioembolic stroke	510	1.03 (0.74–1.45)	0.84	

## Discussion

This retrospective ICU-based study demonstrated that the Aggregate Inflammatory Status Index (AISI), a composite biomarker integrating neutrophils, monocytes, lymphocytes, and platelets, is independently associated with long-term mortality in patients with acute ischemic stroke (AIS), particularly those with large-artery atherosclerotic stroke. AISI exhibited a clear U-shaped association with mortality, suggesting that both excessive inflammation and profound immunosuppression may contribute to poor long-term outcomes.

Inflammation plays a central role in the pathophysiology of AIS [27]. Following ischemia, damage-associated molecular patterns trigger rapid innate immune activation, leading to neutrophil recruitment within hours and peak infiltration at 24–48 h [18, 27–30]. Neutrophils release matrix metalloproteinase-9 and reactive oxygen species, aggravating blood–brain barrier disruption and secondary brain injury [31]. Platelet–leukocyte aggregates further intensify microvascular occlusion and inflammatory activation [32]. Monocytes and macrophages exacerbate injury through the secretion of IL-6 and TNF-α [33]. Whereas lymphocyte subpopulations exert bidirectional effects ranging from IL-10-mediated neuroprotection [34, 35] to IL-17-driven inflammation and tissue damage [36, 37]. Traditional inflammatory markers such as NLR, PLR, and SII have been associated with short-term stroke outcomes [38–41] but remain limited because they capture only partial aspects of immune activation. Because AISI integrates neutrophils, monocytes, lymphocytes, and platelets, it arguably provides a more comprehensive reflection of systemic immune–inflammatory balance. In our cohort, the U-shaped association between AISI and mortality suggests that both excessive inflammation (high AISI) and excessive immunosuppression or immune exhaustion (low AISI) are harmful.

Recent insights into the cholinergic anti-inflammatory pathway (CAP) provide a potential mechanistic explanation for the U-shaped association between AISI and long-term mortality in AIS patients. The CAP is a neuroimmune reflex in which vagal efferent signaling regulates splenic macrophages through α7nAChR to suppress excessive cytokine release [42–44]. Experimental evidence demonstrates that acute ischemic stroke disrupts CAP activity, leading to exaggerated peripheral inflammation, enhanced monocyte and neutrophil activation, and endothelial injury [45]. Conversely, excessive CAP-driven immunosuppression may cause lymphopenia, impaired host defense, and increased susceptibility to post-stroke infection, another major contributor to mortality [46, 47]. Because AISI integrates neutrophils, monocytes, lymphocytes, and platelets, very high AISI levels may reflect CAP failure and hyperinflammation [48], whereas abnormally low AISI may indicate immune exhaustion or excessive neuroimmune suppression [49]. These findings support CAP dysregulation as a plausible biological mechanism underlying the nonlinear prognostic pattern observed with AISI.

In AIS patients, stroke-induced autonomic dysfunction and central nervous system injury may impair CAP signaling, leading to dysregulated systemic inflammation. Under this condition, a high AISI may reflect CAP failure—uncontrolled activation of neutrophils, monocytes, and platelets—leading to endothelial injury, vascular disease progression (e.g., accelerated atherosclerosis), organ damage, or recurrent vascular events, thereby increasing long-term mortality risk. On the other hand, excessively low AISI may indicate over-suppression of immune responses or immune exhaustion mediated by CAP overactivity or compensatory immunosuppression, which may predispose to infections, reduced repair capacity, and long-term complications—again elevating mortality risk. Thus, CAP dysregulation (either loss or overactivity) can produce a nonlinear, *U*-shaped association between systemic inflammatory status (as captured by AISI) and mortality.

Moreover, chronic inflammation and immune imbalance reflected by elevated AISI may promote progression of large-artery atherosclerosis disease through monocyte recruitment, foam cell formation, endothelial dysfunction, and platelet activation [50–52]. This may further worsen long-term vascular health and contribute to increased mortality over time, rather than short-term death, consistent with our finding that AISI did not significantly predict 30-day mortality but was associated with long-term survival.

Overall, our data suggest that AISI captures the dynamic interplay among innate immunity, adaptive immunity, coagulation/inflammation, and neuroimmune regulation, and that CAP may be a key neuroimmunological mechanism underlying the observed prognostic associations. Monitoring AISI in AIS patients may help identify individuals at risk of detrimental immune imbalance and guide potential immunomodulatory interventions aimed at restoring immune homeostasis.

## Limitations

This study has several limitations. First, the study population was derived from a single region, which may have introduced selection or geographic bias; future work will involve a multicenter design to improve generalizability. Second, although numerous clinical covariates are known to influence stroke prognosis, many of these variables were not adequately collected in the present study. In particular, key neurological and functional assessments—such as the National Institutes of Health Stroke Scale (NIHSS), morbidity scores, and quality-of-life measures—were unavailable. In addition, detailed neuroimaging information (e.g., ischemic lesion location and extent) was not recorded, preventing stratified analyses based on stroke severity or lesion distribution. Third, the sample size was relatively small, and larger cohorts are needed to validate our findings. Finally, all-cause mortality was used as the study endpoint. Because the MIMIC-III database does not allow reliable determination of cause-specific mortality, the observed association between AISI and mortality may partly reflect overall disease severity or systemic inflammatory burden rather than stroke-specific fatal outcomes. Therefore, future studies incorporating stroke-related mortality and functional outcomes, such as the modified Rankin scale, are needed for further validation. Future multicenter prospective studies with larger sample sizes and comprehensive clinical and imaging data are warranted to validate these findings.

## Data Availability

The data that support the findings of this study are openly available in [MIMIC-III Clinical Database (version 1.4)] at 10.13026/C2XW26 reference number [Johnson, A., Pollard, T., & Mark, R. (2016). MIMIC-III Clinical Database (version 1.4). PhysioNet. 10.13026/C2XW26.]. The extracted raw data involved in this paper have been placed in the Supplementary Materials.
